# Treatment of critically sized femoral defects with recombinant BMP-2 delivered by a modified mPEG-PLGA biodegradable thermosensitive hydrogel

**DOI:** 10.1186/s12891-016-1131-7

**Published:** 2016-07-15

**Authors:** Kuo-Ti Peng, Meng-Yow Hsieh, Carl T. Lin, Chin-Fu Chen, Mel S. Lee, Yi-You Huang, Pey-Jium Chang

**Affiliations:** Division of Joint Reconstruction, Department of Orthopedic Surgery, Chang-Gung Memorial Hospital, Chiayi, Taiwan; College of Medicine, Chang-Gung University, Taoyuan, Taiwan; Institute of Biomedical Engineering, College of Engineering, College of Medicine, National Taiwan University, No. 1, Sec. 1, Jen-Ai Road, Taipei, Taiwan; Biomedical Technology and Device Research Labs, Industrial Technology Research Institute, Hsinchu, Taiwan; Department of Chemical Engineering, National Tsing-Hua University, Hsinchu, Taiwan; Graduate Institute of Clinical Medical Sciences, College of Medicine, Chang-Gung University, Taoyuan, Taiwan; Department of Nephrology, Chang-Gung Memorial Hospital, Chiayi, Taiwan; Graduate Institute of Clinical Medical Sciences, College of Medicine, Chang-Gung University, 6 West, Chia-Pu Road, Puzi City, Chiayi 613 Taiwan

**Keywords:** Biodegradable polymer, Thermosensitive hydrogel, mPEG-PLGA, Fracture healing, BMP-2

## Abstract

**Background:**

Reconstruction of a segmental fracture with massive bone loss is still a challenge for orthopaedic surgeons. The aim of our study was to develop a suitable biodegradable thermosensitive hydrogel system as a carrier for bone morphogenetic protein (BMP)-2 delivery in the treatment of critical-sized femoral defects.

**Methods:**

A block copolymer composed of monomethoxypoly(ethylene glycol) (mPEG), poly(lactic-co-glycolic acid) (PLGA) and 2, 2’-Bis (2-oxazolin) (Box) was synthesized by ring opening polymerization. The synthesized block copolymer was characterized by ^1^H-NMR spectroscopy and gel permeation chromatography (GPC). Different biophysical and biochemical properties of the synthesized copolymer, including temperature-induced structure changes, degradation rate, pH changes during hydrolytic degradation, cell toxicity, and the release profile of BMP-2, were also evaluated and/or were compared with those of a well-characterized mPEG-PLGA copolymer. In animal testing, rabbits (*n* = 36) that received critically sized (10 mm) femoral defects were divided into 6 groups. These experimental groups included an untreated group, autograft, and groups treated with the synthesized copolymer carrying different concentrations of BMP-2 (0, 5, 10, and 20 μg/ml). Bone repair was evaluated using X-ray radiography, histological staining, micro-computed tomography (μCT), biomarker examination and biomechanical testing in a 12-week treatment period.

**Results:**

A new thermosensitive mPEG-PLGA/Box/mPEG-PLGA block copolymer, or named as BOX copolymer, was successfully prepared. Compared to the reported mPEG-PLGA in vitro, the prepared BOX copolymer at the same weight percent concentrations exhibited wider temperature ranges of gelation, slower degradation rates, higher the pH values, as well as less cytotoxicity. Furthermore, the BMP-2 release from BOX hydrogel exhibited a near-linear release profile in vitro. In animal experiments, treatment of critical-sized bony defects with 25 wt% BOX hydrogel carrying BMP-2 effectively promoted fracture healing during the 12-week trial period and higher concentrations of BMP-2 treatment correlated with better bone quality. Most importantly, clinical outcome and bone healing in the BOX-hydrogel group with 20 μg/ml BMP-2 were nearly equivalent to those in the autograft group in a 12-week treatment course.

**Conclusion:**

These data support that the use of BOX hydrogel (25 wt%) as a drug delivery system is a promising method in the treatment of large bone defects.

## Background

Reconstruction of massive segmental bone defects caused by trauma or tumor is still a significant clinical challenge. Unsatisfactory clinical outcomes occur in more than 30 % of patients with high-energy fracture after surgical treatment [1]. Thus far, there are a variety of interventions available to orthopedic surgeons to manage extensive local bone loss, including autograft, allograft and transplantation with synthetic bone substitutes. Autograft, also known as autologous bone graft, is considered to be a gold standard for bone replacement. Although the use of autograft is considered as the gold standard, some problems are encountered such as donor site morbidity and limited donor bone supply [2, 3]. Allograft is an alternative way for bone regeneration in which the graft bone is obtained from another individual. Several disadvantages have been reported for allograft transplantation, including incomplete or delayed graft incorporation, poor osteoinductivity, the potential for eliciting a deleterious immune response, and the risk of disease transmission [4, 5]. To circumvent these problems of autograft and allograft, various synthetic bone substitutes such as hydroxyapatite, calcium phosphate cements or biodegradable polymers have been developed [6–8]. These synthetic bone substitutes provide the benefits including availability, sterility and reduced morbidity at the graft site.

Theoretically, an ideal bone graft material should include four characteristics: (i) osteointegration, the ability to directly bond to the surface of host bone; (ii) osteoconduction, the ability to serve as a scaffold to guide the growth of bone; (iii) osteoinduction, the ability to stimulate differentiation of osteoprogenitor cells into osteoblasts; (iv) osteogenesis, the formation of new bone by osteoblasts present within the graft material [9]. Based on criteria in the list, only autograft matches all of these requirements, while allograft possesses osteointegrative, osteoconductive and osteoinductive potentials. Currently, synthetic bone substitutes possess only osteointegrative and osteoconductive properties. Due to the lack of osteoinductive and osteogenic abilities, one possible approach to improving synthetic bone substitutes is to incorporate bone morphogenetic protein 2 (BMP-2), the most common cellular osteoinductive mediator that has received FDA approval for the use in treating acute tibial fractures [10]. In addition to exogenous BMP-2 treatment, stem cell-based therapy using mesenchymal stem cells (MSCs) has also received growing attention for bone regeneration [11]. Although MSCs implanted in bone defects are capable of conferring osteogenesis under certain conditions, the potential risks of stem cell-based therapeutic applications are largely unknown [12].

Over the past decade, biodegradable polymers have been extensively studied and used for a wide range of biomedical applications [13]. Among these biodegradable polymers, temperature-responsive hydrogels have attracted great attention as scaffolds for tissue engineering or as carriers for bioactive molecules [14–17]. Compared to other implantable or biodegradable systems, temperature-responsive hydrogels may potentially give equal or greater benefits to treat bone defects. First, the sol-to-gel system provides an injectable design, which allows surgical operation in a minimally invasive way. Second, formulation or preparation of the thermogelling system is free of harmful organic solvents. Third, an irregular-shaped bone defect can easily be filled during the sol-to-gel transition. Fourth, the use of the thermogelling system has a high encapsulation rate for drug or bioactive molecules. Previously, we have developed a biodegradable thermosensitive hydrogel copolymer, the amphiphilic monomethoxypoly (ethylene glycol)-co-poly(lactic-co-glycolic acid) (mPEG-PLGA), which has been used as a drug carrier in treating osteomyelitis [18]. Although the use of mPEG-PLGA copolymer as a drug carrier appears to be an effective approach for the treatment of osteomyelitis, several biochemical properties of mPEG-PLGA copolymer potentially limit its applications in the reconstruction of large bone defects. For examples, the degradation products such as lactic acid and glycolic acid released from mPEG-PLGA hydrogel may lead to a transient pH reduction, which raises concerns regarding local acidosis or inflammation [19, 20]. Another potential drawback is that the degradation rate of mPEG-PLGA hydrogel is fast (50 % within 2 weeks), which is unfavorable if we need to extend drug release for a longer time period.

In the report, we aimed to develop a suitable thermosensitive biodegradable hydrogel for BMP-2 delivery to treat critical-sized bone defect. A modified mPEG-PLGA copolymer that contains an additional linkage of 2, 2’-Bis (2-oxazolin) (Box) was prepared here and was designated as mPEG-PLGA/Box/mPEG-PLGA copolymer or “BOX” hydrogel. Compared to mPEG-PLGA hydrogel in vitro, the BOX hydrogel exhibited wider gelling temperature ranges, slower degradation rates, and higher the pH before and after degradation. In the in vivo examination, BOX hydrogels loaded with different concentrations of BMP-2 were used to treat a critical-sized (10 mm) femoral defect in NZW rabbits. The outcomes of different treated groups were evaluated using X-ray radiography, histological staining, micro-computed tomography (μCT), biomarker examination and biomechanical testing. Our results demonstrated that implantation of the BMP-2-loaded BOX hydrogel is an efficient method for the treatment of femoral bone defects in rabbits.

## Methods

### Chemicals

D,L-lactide and glycolide were purchased from Purac (Netherlands). Poly (ethyleneglycol) monomethyl ether (mPEG) (Mn, 550 g/mol) as an initiator was purchased from Polyscience (Warrington, PA, US). Stannous 2-ethylhexanoate (Stannous Octoate) was obtained from Sigma Aldrich (Poole, UK). Succinic anhydride was purchased from Fluka (St. Louis, MO, US). Recombinant human bone morphogenetic protein 2 (BMP-2) was purchased from Peprotech (Rocky Hill, NJ, US). All other chemicals were used as HPLC grade or extra pure grade.

### Synthesis of BOX copolymer

**Scheme 1:**
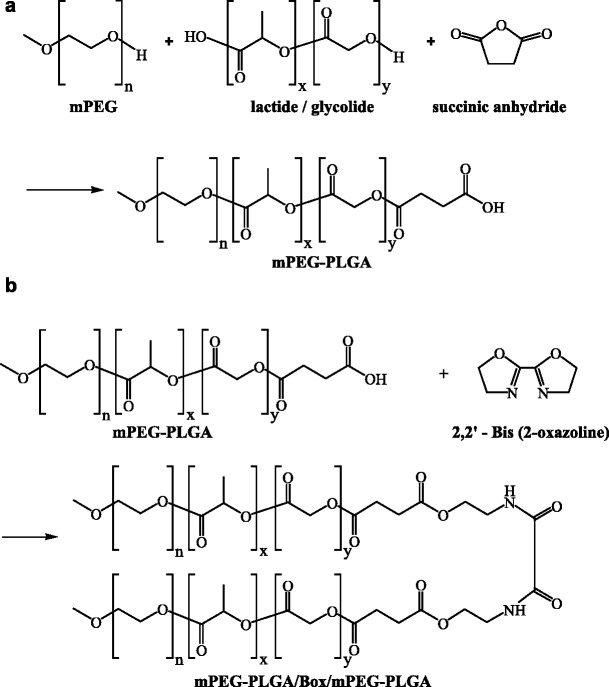
Schematic diagram of the synthesis of BOX hydrogel copolymer

BOX copolymer, also referred to as mPEG-PLGA/Box/mPEG-PLGA, was synthesized by the ring-opening polymerization of monomers. A synthetic procedure is shown in Scheme 1. Briefly, 10.04 g of mPEG, 20 g of lactide, and 5.64 g of glycolide were initially added to the reactor and the temperature was elevated slowly for dissolving the added substances. When the temperature reached and was sustained at 160 °C, 14.0 μl of catalyst (stannous 2-ethyl-hexanoate) was added. After stirring for 8 h, the mPEG-PLGA polymer subunit was obtained. Next, 1.84 g of succinic anhydride (with a molecular weight of 100.07 g/mol) was added into the reactor. After stirring for 4 h, 1.28 g of 2, 2’-Bis (2-oxazolin) (Box) (with a molecular weight of 140.14 g/mol) was added into the reactor. Once the mixture was completely melted, stannous 2-ethylhexanoate as a catalyst was added into the reactor. After polymerizing for 4 h, the product was precipitated with diethyl ether/n-hexane (v/v = 1/9). The resulting copolymers were washed three times and vacuum-dried for 24 h at 40 °C.

### Gel permeation chromatography (GPC)

The molecular weights of the synthetic mPEG-PLGA and BOX copolymers were determined using an Agilent GPC Addon apparatus and a RI-150 refractive index signal detector coupled to Plgel® columns. Tetrahydrofuran served as a chromatographic solvent at 1 ml/min flow rate in the assay and polystyrene standards were used for calibration.

### ^1^H Nuclear Magnetic Resonance (^1^H NMR)

^1^H NMR spectroscopic analysis was performed in CDCl_3_ using an NMR instrument (BRUKER DRX400) at 400 MHz at room temperature. The number-average molecular weight (Mn) and the LA/GA ratio were determined by integration of the signals pertaining to each monomer.

### Determination of sol-gel-sol phase transition by the test tube inverting method

Solutions of hydrogel copolymers in deionized water from 5 to 30 wt% were prepared and stored in vials at 4 °C. After 24 h, the vials containing polymer solutions were immersed in a water bath at 5 °C, and the phase transition of polymer solutions was investigated by raising the bath temperature from 5 to 60 °C in increments of 1.0 °C. The phase transition temperature was taken as the first temperature at which the solution did not flow when tilted.

### Determination of sol-gel-sol phase transition by rheomter

The rheological properties of hydrogel copolymers (in sterile distilled water) were measured using AR-2000EX Rheometer with plate/plate measuring systems and TRIOS application software. The plates were equilibrated to the starting temperature (5 °C) and temperature sweep tests were carried out from 5 to 60 °C. The temperature ramp rate was 0.1 °C/min. Frequency of 1 Hz and the controlled shear strain (1 %) with a deflection angle of 0.5 rad were employed.

### Degradation and pH change of hydrogel copolymers

One milliliter of mPEG-PLGA or BOX hydrogel copolymers (20 wt%) were incubated in 15 ml of phosphate buffered saline (PBS), normal saline (0.9 % NaCl), or distilled water in air-tight vials and the mixed samples were stirred with a speed of 50 rpm at 37 °C. After the indicated incubation periods, the pH of upper layer solution in each sample was measured. Triplicate hydrogel samples were determined at each time point. To determine the degradation of hydrogel copolymers, solid residues in vials were briefly rinsed with deionized water, lyophilized and weighed.

### Cytotoxicity assay

L929 is a murine fibroblast cell line and hFOB1.19 a human fetal osteoblastic cell line. L929 cells were cultured in Eagle’s minimum essential medium supplemented with 10 % horse serum, while hFOB1.19 cells were cultured in a 1:1 mixture of phenol red-free DMEM and Ham’s F-12 medium supplemented with 0.3 mg/ml G418 and 10 % fetal bovine serum. The leachates of mPEG-PLGA and BOX hydrogel copolymers were prepared by using 1 ml of hydrogel copolymers (5–30 wt%) that were incubated with 15 ml of PBS at 37 °C for 24 h. L929 or hFOB1.19 cells (1 × 10^4^) were plated on 96-well plates and cultured in the mixture of the culture medium and copolymer supernatants at a ratio of 1:1 (60 μl: 60 μl). Cell proliferation was evaluated with tetrazolium-based colorimetric assay (XTT assay; Roche). Triplicate samples were examined for each cell line and each experiment was repeated at least three times.

### Intracutaneous irritation test

All animal experimental protocols in the study were approved by the Institutional Animal Care and Use Committee of the Chang Gung Memorial Hospital. The 3-month-old New Zealand White (NZW) rabbits weighing 2.5–3 kg were purchased from the Taiwan Livestock Research Institute, Council of Agriculture, Executive Yuan. The animals were then maintained in the Laboratory Animal Center (ISO9001: 2008) of Chang Gung Memorial hospital at Chiayi. Five intradermal injections of each 0.2 ml BOX hydrogel with 20, 25 and 30 wt% were given to the fur-clipped skin of NZW rabbits. The skin irritation was evaluated at 24, 48 and 72 h after injection.

### BMP-2 release in vitro

The BOX-hydrogel copolymer was sterilized using 15 W UV light for 24 h and recombinant BMP-2 (100 μg) was added into 4 ml of 15, 20, 25 and 30 wt% copolymer aqueous solution. One milliliter of the BMP-2/polymer formulation was loaded into the button of 10 ml release cell and kept at 37 °C for 5 min to form the solid gel, and then 9 ml PBS solution was added to the release cell. The release cell was maintained at 37 °C in a thermostat bath at a shaking rate of 50 rpm. Levels of BMP-2 were determined by reversed-phase HPLC. One milliliter of sample solution was first filtered through 0.2 μm filter and then applied to a HPLC system (JASCO LC-2000) on a reversed-phase column (Hypersil® PEP HS C18, 4.6 × 250 mm, Thermo, US). The concentration of BMP-2 was calculated by external standardization method.

### Creation of critically sized (10 mm) femoral defects

The 3-month-old NZW rabbits, weighing 2.5–3 kg, were used for this study. 36 total rabbits were divided into 6 groups and each group contains 6 rabbits. These 6 groups included an untreated group, autograft, and groups treated with BOX hydrogels containing different concentrations of BMP-2 (0, 5, 10, and 20 μg/ml). All rabbits fasted for 1 day before surgery. Anesthesia was performed with ketamine (25 mg/kg) and xylazine (10 mg/kg) by intravenous injection. The incision was made on the lateral surface and extended down to the middle femur. A MicroHall oscillating saw (Linvatek, Largo, FL) was used to create a 10 mm length bony defect in the middle shaft of the femur. The osteotomized femur was stabilized with a stainless plate (DC-Plate, Synthes, Davos, Switzerland), screws and multiple looping wires. Implant materials including autologous bone and 1 ml of BOX hydrogels with or without BMP-2 were inserted into the segmental defects at the right femora of NZW rabbits. As noted, autologous bones (10 mm) were cut into small pieces (2–3 mm) using a bone rongeur and then re-implanted into the defect site.

### Radiographic analysis and μCT

The X-ray images were analyzed using the PMOD software (PMOD Technologies, Zu rich, Switzerland) to define areas of the occupying callus and the original defect. Based on the X-ray radiography, the bone was considered healed if i) contiguous calluses spanning both proximal and distal ends of bony defects were observed, and ii) the area of the newly formed bone exceeded 25 % of the defect area. After 12 weeks, rabbits were sacrificed and all femora were scanned with an animal μCT imaging system (Skyscan 1076, Bruker, Belgium). Bone density in the osteotomized site with 10 × 10 mm area was measured and calculated by μCT software provided in the system (CT Analyser, Bruker, Belgium).

### Histological staining

Femoral bone samples were fixed in neutral-buffered formalin and decalcified with 0.6 N HCl. A 3-cm segment in the mid-shaft of the diaphysis (encompassing the graft) was cut, embedded in paraffin and sectioned longitudinally (8 mm thick). The sections from the mid-defect region were stained with hematoxylin and eosin (H&E).

### Biomechanical testing

The strength of femur specimens from all rabbit samples (*N* = 36) in 6 treatment groups was evaluated by destructive biomechanical testing. The proximal and distal ends of specimens were embedded in polymethylmethacrylate blocks and rigidly mounted on a universal testing machine (MTS; Minneapolis, MN). The distal end of the specimen was rotated laterally at a constant deformation rate of 1.0° per minute until bone failure occurred. The maximum torque at failure (N-mm), the angle at failure (deg) and torsional stiffness (N-mm/deg) were computed according to the applied load and angular displacement curves.

### Western blot analysis

Bone specimens were homogenized and lysed in a protein extraction reagent (Tissue protein extraction reagent, Pierce, Rockford, US) containing protease inhibitors (Protease Inhibitor cocktail, Sigma-Aldrich, US). Western blot analysis was carried out according to our previous publication [18]. Antibodies specific to collagen I (COL1A1; ab34710, Abcam, UK), osteocalcin (OC; ab13418, Abcam, UK), and alkaline phosphatase (ALP; sc-28904, Santa Cruz, US) were used at 1:1000. The HRP-conjugated anti-mouse and anti-rabbit secondary antibodies, (Chemicon, US) were used at 1:5000. After extensive washing steps, the membranes were incubated with ECL substrates (Millipore, US).

### Statistical analysis

A Student’s *t*-test was used to compare the biophysical and biochemical properties between mPEG-PLGA and BOX copolymers. The differences of clinical outcomes or biomarker expression between the untreated group and treated groups were statistically analyzed using Kruskal Wallis (KW) test. *P* < 0.05 was considered to be statistically significant.

## Results

### Preparation of BOX copolymer

**Fig. 1:**
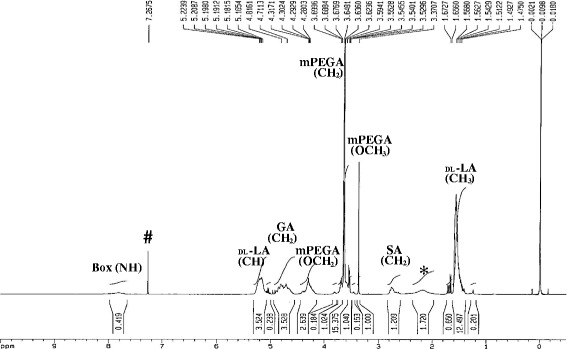
^1^H NMR spectrum of BOX copolymer plotted as signal intensity (vertical axis) vs. chemical shift (in ppm on the horizontal axis). * H_2_O; # CDCl_3_

BOX copolymer, or mPEG-PLGA/Box/mPEG-PLGA, is synthesized as shown in Scheme 1 and the ^1^H NMR spectrum of BOX copolymer is given in Fig. 1. The complicated splits in these peaks are due to random copolymerization of glycolide (GA) and lactide (LA). The characteristic signals appearing at 7.8, 5.2, 4.8, 4.2, 3.6, 3.3, 2.6 and 1.5 ppm represent NH of 2, 2’-Bis (2-oxazolin) (Box), CH of LA, CH_2_ of GA, CH_2_O of mPEG, CH_2_ of mPEG, OCH_3_ of mPEG, CH_2_ of SA, and CH_3_ of LA, respectively. The LA-to-GA ratio determined by ^1^H NMR spectroscopy matched very well with the initial ratio (78:22) of the added monomers. As expected, the calculated molecular weight averages (Mw and Mn) of BOX copolymer were more than twice (2.37 to 2.40) as high as those of the parental mPEG-PLGA copolymer (Table 1). Furthermore, a narrow molecular weight distribution (MWD; Mw/Mn) around 1.38 to 1.40 was obtained in both copolymers (Table 1).

**Table 1:** The molecular weight averages and molecular weight distribution of mPEG-PLGA and BOX hydrogel copolymers

Thermosensitive Copolymer	Molecular weight		
	Mw^a^	Mn^b^	MWD^c^
mPEG-PLGA hydrogel	2257	1635	1.38
BOX hydrogel	5415	3867	1.40

^a^Mw: The weight average molecular weight

^b^Mn: The number average molecular weight

^c^MWD: Molecular weight distribution

### Thermosensitive sol-gel-sol transition of mPEG-PLGA and BOX copolymers

**Fig. 2:**
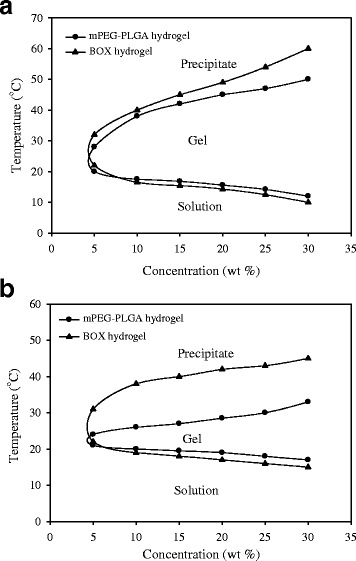
Sol-to-gel-to-sol phase diagrams of mPEG-PLGA and BOX hydrogel copolymers in deionized water. The phase transition temperatures were measured by the tube inverting method (**a**) and by rheometer (**b**)

To compare the sol-to-gel-to-sol behaviors between BOX and mPEG-PLGA copolymers, the phase transition was determined by both the test tube inverting method and rheometer. Aqueous solutions of copolymers at concentrations from 5 to 30 wt% were first prepared in deionized water (4 °C). All samples of both copolymers were shown as a translucent emulsion solution at 4 °C. The phase transition diagrams of both copolymers determined by the test tube inverting method are shown in Fig. 2a. Both hydrogel copolymers exhibited three physical states: solution, gel, and precipitate in responding to different temperatures. When the copolymer concentrations were gradually increased from 5 to 30 wt%, the solution-to-gel (sol-gel) transition temperatures were gradually decreased and the gel-to-precipitate (gel-sol) transition temperatures were raised. The critical gelation concentrations (CGCs) of the mPEG-PLGA-hydrogel and BOX-hydrogel copolymers were estimated to be 4.0 wt% (Fig. 2a, tips of the U curves). Based on the testing, BOX hydrogel showed similar the sol-gel transition temperatures to mPEG-PLGA hydrogel, but had higher the gel-sol transition temperatures than mPEG-PLGA hydrogel.

When the sol-gel-sol transition of both mPEG-PLGA and BOX hydrogels was determined by rheometer (Fig. 2b), we found that the gel-sol transition temperatures measured by the test tube inverting method or by rheometer could vary by 40 %. Noteworthily, the gel-sol transition temperatures of BOX copolymer measured by rheomter were much higher (up to 10 °C) than those of mPEG-PLGA copolymer.

In addition to the sol-gel-sol transition described above, we also compared the gelling time of mPEG-PLGA and BOX copolymers with different concentrations (15, 20, 25 and 30 wt%) at 37 °C (Table 2). In general, the gelation times of BOX solutions were relatively shorter than those of mPEG-PLGA solutions (32–145 s versus 43–163 s).

**Table 2:** Gelling time for hydrogel (seconds)

	mPEG-PLGA hydrogel (mean ± SD)	BOX hydrogel (mean ± SD)
15 wt%	163 ± 7	145 ± 6
20 wt%	98 ± 5	95 ± 5
25 wt%	79 ± 5	58 ± 4
30 wt%	43 ± 3	32 ± 2

### In vitro degradation of mPEG-PLGA and BOX hydrogels

**Fig. 3:**
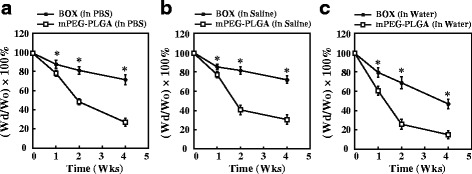
Degradation of mPEG-PLGA and BOX hydrogels in vitro. Both mPEG-PLGA and BOX hydrogels at a concentration of 20 wt% were immersed in 15 ml of PBS (**a**), normal saline (**b**) or distilled water (**c**). The mass loss of both copolymers was measured at 37 °C in a 4-week incubation period. *P < 0.05, the statistical comparisons for BOX hydrogel versus mPEG-PLGA hydrogel

Both mPEG-PLGA and BOX hydrogels copolymers with 20 wt% were immersed in PBS, normal saline or water at 37 °C, and the weight loss of both copolymers was measured within 4 weeks (Fig. 3). We found that mPEG-PLGA and BOX hydrogels in PBS or in normal saline had slower degradation rates than those in water (Fig. 3), suggesting that physiological or chemical buffering systems could slow down the degradation of these two copolymers. Under the same conditions, BOX hydrogels exhibited slower degradation than mPEG-PLGA hydrogels. For example, mPEG-PLGA and BOX hydrogels in PBS or in normal saline were degraded by 70 and 30 %, respectively, at 4-week incubation.

### pH changes in mPEG-PLGA and BOX hydrogels during hydrolytic degradation

**Fig. 4:**
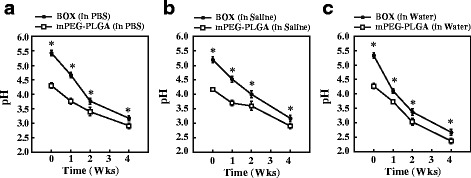
Changes in the pH of mPEG-PLGA and BOX hydrogels before and after degradation. The pH of mPEG-PLGA and BOX hydrogel copolymers was measured after immersion in PBS (**a**), saline (**b**) or distilled water (**c**) for 0, 1, 2 and 4 weeks. *P < 0.05, the statistical comparisons for BOX hydrogel versus mPEG-PLGA hydrogel

Due to the presence of the basic 2,2’-Bis(2-oxazoline) group in the BOX copolymer, the prepared BOX hydrogel displayed higher the pH than the corresponding mPEG-PLGA hydrogel before hydrolytic degradation (pH 5.4 versus pH 4.2). After incubation with PBS, normal saline or water, both mPEG-PLGA and BOX copolymers underwent hydrolytic degradation and pH values were gradually decreased over time (Fig. 4). Although pH values were reduced for both copolymers after hydrolytic degradation, BOX hydrogel consistently showed pH values higher than those of mPEG-PLGA hydrogel during the 4-week trial period.

### Cytotoxicity of mPEG-PLGA and BOX hydrogels

**Fig. 5:**
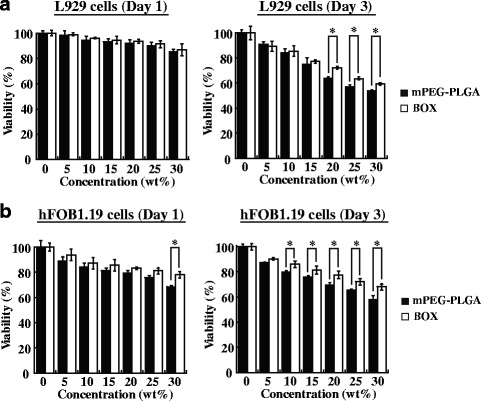
In vitro cytotoxicity of mPEG-PLGA and BOX hydrogel copolymers. The leachates of mPEG-PLGA or BOX hydrogels (5–30 wt%) were prepared as described in Materials and Methods. L929 cells (**a**) and hFOB1.19 cells (**b**) were cultured for 1 or 3 days in the 1:1 mixed medium of culture media and copolymer leachates. Cell proliferation was measured by XTT assay. *P < 0.05, the statistical comparisons for BOX-hydrogel treatment versus mPEG-PLGA-hydrogel treatment

To test cytotoxicity of mPEG-PLGA and BOX copolymers in vitro, L929 mouse fibroblasts and hFOB1.19 human osteoblastic cells were included in the study. Both L929 and hFOB1.19 cells were cultured in a 1:1 mixture of the culture medium and the leachates of copolymers (5 to 30 wt%). Proliferation of the cultured cells was evaluated on day 1 and day 3 by XTT assay. There were no obvious differences between mPEG-PLGA- and BOX-treated groups in either L929 or hFOB1.19 cells on day 1 (Fig. 5). However, at day 3 of the culture, we found that the leachates of BOX copolymer at high-concentration formulations (>15 wt%) were less toxic than those of mPEG-PLGA copolymer on both L929 and hFOB1.19 cells (Fig. 5).

**Fig. 6:**
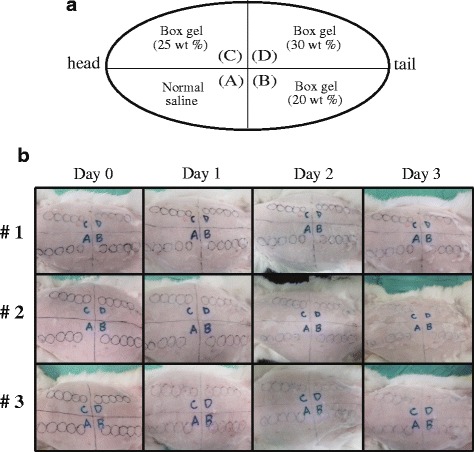
Intracutaneous irritation test. (**a**) Diagram of arrangement of injection sites for tested samples (including normal saline and the BOX hydrogel at 20, 25 and 30 wt%) in rabbits. (**b**) Five intradermal injections of each 0.2 ml normal saline or the BOX hydrogel with different formulations (20, 25 and 30 wt%) were given to the fur-clipped skin of rabbits (*N* = 3). The skin irritation was evaluated at day 1, 2 and 3 after injection

To further determine the toxicity of BOX hydrogel copolymer in vivo, the intracutaneous irritation test was carried out in rabbits. BOX hydrogels with 20, 25 and 30 wt% formulations were intracutaneously injected (0.2 ml per site) into rabbits and skin irritation was evaluated at day 1, 2 and 3 after injection. We found that injection of the BOX hydrogel in rabbits did not show any signs of skin irritation over 3 days (Fig. 6).

### BMP-2 release from BOX hydrogel in vitro

**Fig. 7:**
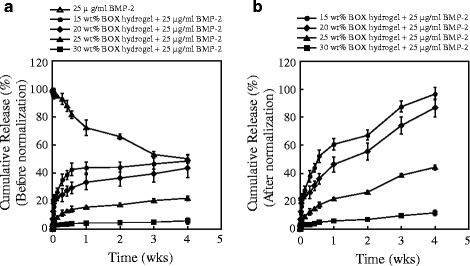
The release profiles of BMP-2 from BOX hydrogels with different copolymer concentrations. **a** The amounts of BMP-2 released from 15, 20, 25 and 30 wt% BOX hydrogels at indicated incubation time points were determined by reversed-phase HPLC. Soluble BMP-2 in the solution (PBS) for different time periods was also included in the study. **b** The release profiles of BMP-2 were plotted after the amounts of BMP-2 released from BOX hydrogels were normalized with the amounts of free BMP-2 in the solution at the corresponding time points

The release profiles of BMP-2 (25 μg/ml) from BOX hydrogels at different copolymer concentrations (15, 20, 25 and 30 wt%) in vitro are shown in Fig. 7. Lower concentrations of copolymer appeared to release more BMP-2 within a 4-week incubation period. At 4 weeks, the measured amounts of BMP-2 released from 15, 20, 25 and 30 wt% Box hydrogels were 48, 43, 22 and 6 %, respectively (Fig. 7a). In parallel, we observed that free BMP-2 was unstable in the solution and up to 50 % of BMP-2 underwent proteolytic degradation at 37 °C after 4-week incubation (Fig. 7a, BMP-2 alone). After normalization of the measured amounts of BMP-2 released from hydrogels to the amounts of free BMP-2 in the solution at the indicated time points, a nearly linear BMP-2 release from BOX hydrogels was obtained (Fig. 7b). The normalized BMP-2 release rate from BOX hydrogels at 4 weeks was about twice as high as the unnormalized BMP-2 release rate shown in Fig. 7a.

### Treatment of critical-sized bone defects with the BMP2-loaded BOX hydrogel

**Fig. 8:**
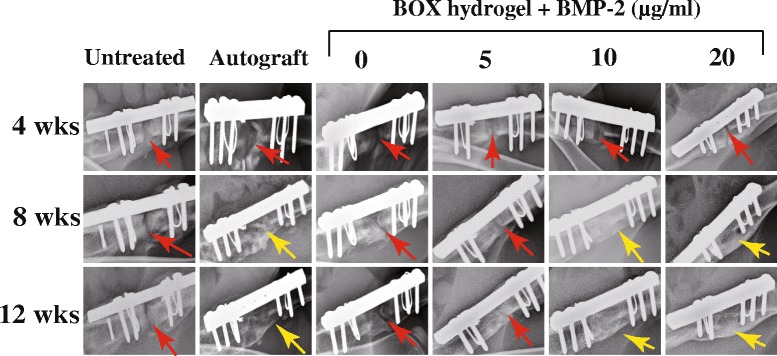
Representative radiographs of untreated and treated femora in rabbits with a critical-sized bone defect. Rabbits that received a 10 mm-long bone defect at the middle of the femur were untreated or treated with autograft bone or BOX hydrogels loaded with different concentrations of BMP-2. The untreated and treated femora at 4, 8 and 12 weeks after operation were examined by X-ray radiography. Red arrows indicate bony defect sites at the femur, and yellow arrows show bridging callus

Based on the BMP-2 release profiles shown in Fig. 7, we chose 25 wt% BOX hydrogel for BMP-2 delivery to treat fracture nonunions in rabbits. One milliliter of the BOX hydrogel alone or the BOX hydrogel loaded with 5, 10 or 20 μg of BMP-2 was injected into the femoral osteotomy gap (10 mm) in the rabbit model. In addition to BOX-hydrogel groups, two control groups including autograft and the untreated group were also included in the study. At 4, 8 and 12 weeks after treatment, bone healing was visualized by X-ray radiography. The representative radiographs are shown in Fig. 8. Radiographic healing did not occur in both the untreated (0/6) and BMP-2-free hydrogel groups (0/6) at 8 and 12 weeks (Table 3). Treatment with the BOX hydrogel containing the low dose of BMP-2 (5 μg/ml) healed some bone defect with a radiographic healing rate 16 % (1/6) and 33 % (2/6) at 8 and 12 weeks, respectively. Bone regeneration was further enhanced in the BMP-2 (10 μg/ml)-loaded hydrogel group with a healing rate 50 % (3/6) at 8 weeks and 67 % (4/6) at 12 weeks. Both the autograft and high-dose BMP-2 (20 μg/ml) treated groups demonstrated 100 % healing rate (6/6, and 6/6) at 8 and 12 weeks (Table 3).

**Table 3:** Radiographic assessment of bone defect healing in rabbits

Groups	Number of animals with radiographic healing	
	8 weeks	12 weeks
Untreated	0/6	0/6
Autograft	6/6	6/6
BOX hydrogel	0/6	0/6
BOX hydrogel + 5 μg/ml BMP-2	1/6	2/6
BOX hydrogel + 10 μg/ml BMP-2	3/6	4/6
BOX hydrogel + 20 μg/ml BMP-2	6/6	6/6

**Fig. 9:**
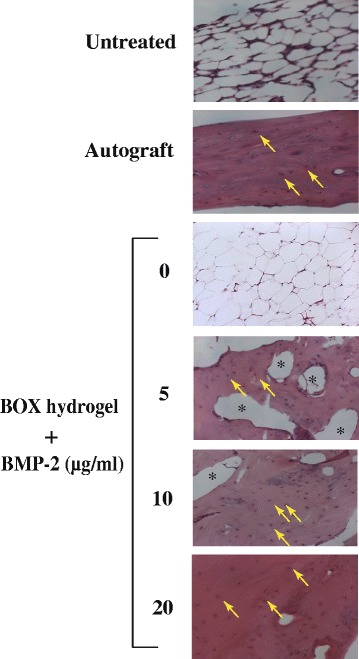
Histological analysis (H&E stain, 200x) of bone regeneration in femoral defect rabbits after treatment. The tissue sections from the mid-defect femur regions of rabbits were prepared and stained with hematoxylin and eosin. Homogenous red staining: bone collagen; arrows: osteocyte; asterisks: femoral bone cavities

To further verify bone healing of the nonunion after BMP-2 treatment, sample sections were subjected to hematoxylin and eosin (H&E) staining (Fig. 9). Three BMP-2-loaded hydrogel groups displayed better bone regeneration than the untreated and BMP-2-free hydrogel groups. Especially, the hydrogel group with the high-dose BMP-2 (20 μg/ml) offered bone healing more effectively than the other BMP-loaded hydrogel groups, and the resulting bone growth was comparable to that in the autograft group (Fig. 9).

### Evaluation of bone repair by the examination of bone remodeling markers

**Fig. 10:**
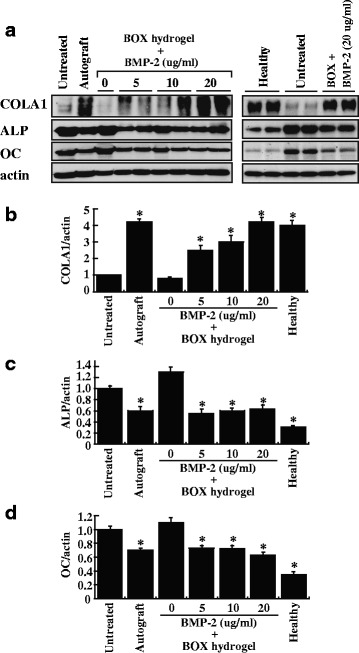
Expression of bone regeneration markers in femoral defects after treatment. **a** After 12-week treatments, the indicated samples were examined for the expression of COL1A1, ALP and OC by Western blot analysis. After normalization with the β-actin content, the relative expression levels of COL1A1, ALP and OC were shown in (**b**, **c** and **d**). Healthy: healthy femora, *P < 0.05 compared to the untreated group

The expression of bone-specific turnover markers, including alpha-1 type I collagen (COL1A1), alkaline phosphatase (ALP) and osteocalcin (OC), was evaluated in the nonunion animal model after treatments. In parallel, bone samples obtained from healthy femora were also included as positive controls (Fig. 10a, *right panel*). When compared to the untreated and BMP-2-free hydrogel groups, the autograft and BMP-2-loaded hydrogel groups showed higher expression levels of COL1A1 (Fig. 10a and b; *P* < 0.05). As expected, the protein abundance of COL1A1 in these treated groups positively correlated with the bone healing detected by X-ray radiography and by H&E staining (Fig. 10a). We also found that, like healthy bone controls, bone samples from the autograft and BMP-2-loaded hydrogel groups consistently had lower expression levels of ALP and OC, two early markers of osteoblastic differentiation, than the untreated and BMP-2-free hydrogel groups (Fig. 10a, c and d; *P* < 0.05).

### Bone healing evaluated by gross appearance and μCT analysis

**Fig. 11:**
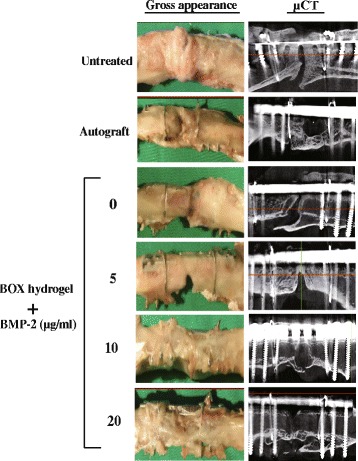
Bone healing evaluated by gross appearance and μCT analysis

At 12 weeks after treatment, rabbits were sacrificed and the femora were prepared for macroscopic observation and μCT bone analysis. The untreated and BMP-2-free hydrogel groups were filled with soft tissues in the defects according to gross appearance (Fig. 11), and the callus did not bridge across the defective areas in these two groups as confirmed by μCT images (Fig. 11). Treatment with BOX hydrogels carrying low or medium BMP-2 (5 μg/ml or 10 μg/ml) promoted the formation of contiguous callus spanning the ends of the bony defect, but not yet fully ossified. Importantly, the complete cortical bone healing was observed in the hydrogel group loaded with 20 μg/ml BMP-2 and in the autograft group. Table 4 shows the bone density of different treated groups measured by μCT.

**Table 4:** Bone mineral density (in grams per square centimeter, g/cm^2^)

Groups	Mean ± SD	*p*
Untreated	0.37 ± 0.03	
Allograft	0.80 ± 0.09	0.000^a^
BOX hydrogel	0.41 ± 0.05	0.140
BOX hydrogel + 5 μg/ml BMP-2	0.61 ± 0.06	0.001^a^
BOX hydrogel + 10 μg/ml BMP-2	0.73 ± 0.05	0.000^a^
BOX hydrogel + 20 μg/ml BMP-2	0.90 ± 0.08	0.000^a^

^a^compared to the untreated group

### Biomechanical tests of bone healing

Tables 5 and 6 show the torsional stiffness and maximum torque of all femora obtained from different treated groups. The untreated or BMP-2-free hydrogel group displayed extremely low torsional stiffness and maximum torque relative to those of the autograft and BMP-2-loaded hydrogel groups. Compared to autograft, the BOX-hydrogel groups loaded with 5, 10 and 20 μg/ml of BMP-2 had a similar torsional stiffness (174.5, 182.2 and 223.3 N-mm/deg versus 200.8 N-mm/deg) and maximum torque (733, 770.8 and 909.3 N-mm versus 713 N-mm). Our results revealed that the thermosensitive BOX hydrogel carrying BMP-2 represents an effective method for repairing large bone defects.

**Table 5:** Torsional stiffness (N-mm/deg)

	Untreated (*n* = 6)	Autograft (*n* = 6)	BOX hydrogel + BMP-2 (μg/ml) (*n* = 6)			
			0	5	10	20
	3	225	16	132	262	302
	6	182	0	210	205	250
	18	150	5	238	240	223
	0	272	6	210	154	163
	5	120	5	112	124	242
	8	280	2	145	108	160
Mean ± SD	6.7 ± 5.6	200.8 ± 59.6^a^	5.7 ± 5.1	174.5 ± 46.8^a^	182.2 ± 57.6^a^	223.3 ± 49.8^a^

^a^ *P* < 0.05 compared to the untreated group. *n* = 6 in each group

**Table 6:** Maximum torque (N-mm)

	Untreated (*n* = 6)	Autograft (*n* = 6)	BOX hydrogel + BMP-2 (μg/ml) (*n* = 6)			
			0	5	10	20
	30	588	38	565	798	1215
	26	750	15	885	695	995
	36	665	55	750	1006	685
	10	840	26	852	658	598
	26	780	31	648	612	1305
	18	655	26	698	856	658
Mean ± SD	24.3 ± 8.4	713.0 ± 84.9^a^	31.8 ± 12.4	733.0 ± 111.2^a^	770.8 ± 133.5^a^	909.3 ± 279.2^a^

^a^ *P* < 0.05 compared to the untreated group. *n* = 6 in each group

## Discussion

Thus far, repair of large bone defects still remains a challenge for orthopaedic surgeons. Studies have reported that natural or synthetic materials could serve as bone graft substitutes or as drug delivery vehicles in bone tissue regeneration [21–25]. Although many advantages of these materials have been reported, an easy-to-prepare biomaterial with desired properties still remains to be developed. In the study, we develop a new thermosensitive hydrogel, named as “BOX”, for BMP-2 delivery to treat fracture nonunions in rabbits. To our knowledge, this is the first report to apply a thermosensitive hydrogel as a BMP-2 carrier to treat large bone defects. In our critical-sized bone defect model, we show that the BOX hydrogel carrying BMP-2 is an effective strategy to treat the nonunion and the resultant bone healing can be comparable to that treated with autologous bone grafting.

mPEG-PLGA hydrogel has previously been developed as a carrier for antibiotics to treat osteomyelitis [18]. Different biophysical and biochemical properties between mPEG-PLGA and BOX hydrogels in vitro have been compared in the study (Figs. 2, 3, 4 and 5). There are at least five reasons that prompted us to choose BOX hydrogel, but not mPEG-PLGA hydrogel, as a BMP-2 carrier for the treatment of fracture nonunions. First, BOX hydrogel has a wider temperature range for gelation than the corresponding mPEG-PLGA hydrogel (Fig. 2), indicating that BOX hydrogel would be more stable than mPEG-PLGA hydrogel in the body. Second, BOX hydrogel has a slower degradation rate than mPEG-PLGA hydrogel under physiological and chemical buffering systems (Fig. 3). The degradation rate of BOX hydrogel (30 % in 4 weeks) is suitable for the treatment of nonunion in a 12-week course. Third, BOX hydrogel exhibits higher the pH than mPEG-PLGA hydrogel during hydrolytic degradation (Fig. 4). Despite the fact that acidic degradation products (lactic acid and glycolic acid) released from mPEG-PLGA or BOX hydrogel could be easily metabolized in the body, transient pH reduction at local implant sites is still the concern for the use of PLGA-based copolymers in vivo [19, 20]. Due to the presence of a basic 2,2’-Bis(2-oxazoline) group, BOX hydrogel consistently displays higher the pH values than mPEG-PLGA. Although BOX hydrogel is still slightly acidic (pH5.4), several studies have previously reported that mild acidic conditions were actually favorable for the osteoinductive activity of BMP-2 in vivo [21, 26]. Fourth, BOX hydrogel is less toxic to cultured cells than mPEG-PLGA hydrogel (Fig. 5). Fifth, a near-linear release profile of BMP-2 from the BOX hydrogel could be achieved over a 4-week incubation period (Fig. 7). Herein, we chose 25 wt% BOX hydrogel in our animal experiments.

In addition to BMP-2-loaded BOX-hydrogel groups, three experimental control groups were also covered in the study, including an untreated group, autograft, and a BMP2-free hydrogel group. In a 12-week treatment course, bone healing was evaluated using X-ray radiography, histological staining, micro-computed tomography (μCT), biomarker examination and biomechanical testing (Figs. 8, 9, 10 and 11 and Tables 3,  4,  5 and 6). According to our results, treatment with BOX hydrogel carrying higher doses of BMP-2 closely correlated with better fracture healing in rabbits. The dose-dependent therapeutic effects strongly suggested that the selected BOX formulation (25 wt%) is suitable to act as a drug carrier for BMP-2 delivery in the animal model. Particularly, treatment of critical-sized bone defects with the BOX hydrogel carrying 20 μg of BMP-2 exhibits excellent outcomes that are similar to those seen in rabbits treated with autologous bone graft.

When the biomarkers of bone formation were examined, we found that differential expressions of COL1A1, ALP and OC were detected in different treatment groups (Fig. 10). As expected, the expression levels of COL1A1 were correlated with bone healing in these treated groups (Fig. 10a and b). However, the levels of ALP and OC were inversely associated with bone healing in our study (Fig. 10a, c and d). COL1A1, ALP and OC are mainly expressed from osteoblasts. During the healing of bone fracture, osteoprogenitor cells from surrounding tissues initially differentiate into osteoblasts, which play a vital role for bone formation and repair. Upon full healing of the bone defect, osteoblasts in bone matrix will transform into osteocytes, the most common cells in mature bone. Therefore, the optimal expression of OC and ALP is believed to occur early during osteoblastic differentiation, but down-regulation of these two markers will be seen after the bone healing is complete. It is consistent with our findings that healthy bones had higher COL1A1 levels but lower ALP and OC levels than broken bones that left untreated (Fig. 10). According to the expression profile of these biomarkers in these treated groups (Fig. 10), we conclude that low amounts of COL1A1 in combination with high amounts of ALP and OC in the untreated group and in the BMP2-free hydrogel group reflect a state of incomplete bone healing.

## Conclusion

The temperature-responsive BOX copolymer, or mPEG-PLGA/Box/mPEG-PLGA, has been successfully prepared and characterized. Based on the in vitro and in vivo testing, we suggest that the use of the thermosentive BOX hydrogel with BMP-2 may hold promise as an alternative therapeutic strategy for the treatment of large bone defects.

## Abbreviations

ALP, alkaline phosphatase; BMP-2, bone morphogenetic protein-2; Box, 2, 2’-Bis (2-oxazolin); BOX, mPEG-PLGA/Box/mPEG-PLGA; COL1A1, alpha-1 type I collagen; GPC, gel permeation chromatography; mPEG, monomethoxypoly(ethylene glycol); mPEG-PLGA, monomethoxypoly (ethylene glycol)-co-poly(lactic-co-glycolic acid); MSCs, mesenchymal stem cells; NMR, nuclear magnetic resonance; OC, osteocalcin; PBS, phosphate buffered saline; PLGA, poly(lactic-co-glycolic acid); μCT, micro-computed tomography
